# Detecting network states in white noise

**DOI:** 10.1186/1471-2202-16-S1-P92

**Published:** 2015-12-18

**Authors:** Jaroslav Hlinka, Michal Hadrava

**Affiliations:** 1 Institute of Computer Science, Czech Academy of Sciences, Prague, Czech Republic; 2 Faculty of Electrical Engineering, Department of Cybernetics, Czech Technical University in Prague, Prague, Czech Republic

## 

Nonstationarity of neural dynamics is a ubiquitous property that is crucial to understanding many key phenomena of both healthy and diseased brain function, including circadian rhythms, dynamics of epileptic activity as well as cognitive processing. Detecting switching of brain states has recently become of growing interest in the human brain neuroimaging community. However, from the data analysis/modelling perspective the task is quite challenging, and competing approaches exist [1]. One widely adopted approach is the use of clustering methods in the temporal domain to detect temporally contiguous clusters of time points with a similar structure of some instantaneous property - e.g. neural activity or functional connectivity profile. While this approach may in principle help to explore the switching structure of brain dynamics, it comes with technical challenges related the presence of noise in both the dynamics and measurements. In particular, as we documented in a recent study [2], comparison of the results with an appropriate null hypothesis is necessary to avoid spurious detection of nonstationarity markers such as switching of neural network states.

We document this danger by applying an example analysis pipeline used in [3] to simulated EEG datasets. The simulated data are generated as realizations of temporally white noise process (either spatially uncorrelated or spatially correlated in a pattern corresponding to real EEG data). In each case, one hundred realizations of a 5 seconds long epoch of N = 20 'electrodes' (each of 2500 time points corresponding to 2ms sampling rate). A k-means clustering algorithm with k = 2 to 10 is applied to cluster the instantaneous synchronization likelihood matrix estimates with parameters as in [3]. The key observation stable across all setting is that the applied typical network switching analysis pipeline leads to spurious discovery of a multitude of network states in the stationary process realizations, with dominant state duration timescales of several tens to hundreds milliseconds qualitatively similar to the original results reported in [3]. These results suggest that observations of network switching should be always cautiously interpreted and tested against appropriate null models.
